# Comparison of 3 Safety-Net Hospital Definitions and Association With Hospital Characteristics

**DOI:** 10.1001/jamanetworkopen.2019.8577

**Published:** 2019-08-07

**Authors:** Ioana Popescu, Kathryn R. Fingar, Eli Cutler, Jing Guo, H. Joanna Jiang

**Affiliations:** 1 Division of General Internal Medicine and Health Services Research, David Geffen School of Medicine at UCLA, Los Angeles, California; 2 Rand Corporation, Los Angeles, California; 3 IBM Watson Health, Sacramento, California; 4 currently with Qventus, San Jose, California; 5 Agency for Healthcare Research and Quality, Rockville, Maryland

## Abstract

**Importance:**

No consensus exists on how to define safety-net hospitals (SNHs) for research or policy decision-making. Identifying which types of hospitals are classified as SNHs under different definitions is key to assessing policies that affect SNH funding.

**Objective:**

To examine characteristics of SNHs as classified under 3 common definitions.

**Design, Setting, and Participants:**

This cross-sectional analysis includes noncritical-access hospitals in the Healthcare Cost and Utilization Project State Inpatient Databases from 47 US states for fiscal year 2015, linked to the Centers for Medicare & Medicaid Services Hospital Cost Reports and to the American Hospital Association Annual Survey. Data were analyzed from March 1 through September 30, 2018.

**Exposures:**

Hospital characteristics including organizational characteristics, scope of services provided, and financial attributes.

**Main Outcomes and Measures:**

Definitions of SNH based on Medicaid and Medicare Supplemental Security Income inpatient days historically used to determine Medicare Disproportionate Share Hospital (DSH) payments; Medicaid and uninsured caseload; and uncompensated care costs. For each measure, SNHs were defined as those within the top quartile for each state.

**Results:**

The 2066 hospitals in this study were distributed across the Northeast (340 [16.5%]), Midwest (587 [28.4%]), South (790 [38.2%]), and West (349 [16.9%]). Concordance between definitions was low; 269 hospitals (13.0%) or fewer were identified as SNHs under any 2 definitions. Uncompensated care captured smaller (200 of 523 [38.2%]) and more rural (65 of 523 [12.4%]) SNHs, whereas DSH index and Medicaid and uncompensated caseload identified SNHs that were larger (264 of 518 [51.0%] and 158 of 487 [32.4%], respectively) and teaching facilities (337 of 518 [65.1%] and 229 of 487 [47.0%], respectively) that provided more essential services than non-SNHs. Uncompensated care also distinguished remarkable financial differences between SNHs and non-SNHs. Under the uncompensated care definition, median (interquartile range [IQR]) bad debt ($27.1 [$15.5-$44.3] vs $12.8 [$6.7-$21.6] per $1000 of operating expenses; *P* < .001) and charity care ($19.9 [$9.3-$34.1] vs $9.1 [$4.0-$18.7] per $1000 of operating expenses) were twice as high and median (IQR) unreimbursed costs ($32.6 [$12.4-$55.4] vs $23.6 [$9.0-$42.7] per $1000 of operating expenses; *P* < .001) were 38% higher for SNHs than for non-SNHs. Safety-net hospitals defined by uncompensated care burden had lower median (IQR) total (4.7% [0%-9.9%] vs 5.8% [1.2%-11.2%]; *P* = .003) and operating (0.3% [−8.0% to 7.2%] vs 2.3% [−3.9% to 8.9%]; *P* < .001) margins than their non-SNH counterparts, whereas differences between SNH and non-SNH profit margins generally were not statistically significant under the other 2 definitions.

**Conclusions and Relevance:**

Different SNH definitions identify hospitals with different characteristics and financial conditions. The new DSH formula, which accounts for uncompensated care, may lead to redistributed payments across hospitals. Our results may inform which types of hospitals will experience funding changes as DSH payment policies evolve.

## Key Points

**Question:**

Does a safety-net hospital definition based on uncompensated care identify different types of hospitals compared with other definitions?

**Findings:**

In this cross-sectional study of 2066 hospitals, the Medicare Disproportionate Share Hospital index identified larger teaching safety-net hospitals, whereas a definition based on uncompensated care captured smaller, rural safety-net hospitals at greater financial risk. Bad debt and charity care were approximately twice as high for safety-net hospitals as for non–safety-net hospitals, unreimbursed costs were 38% higher, and operating margins were more than 6 times lower.

**Meaning:**

Medicare Disproportionate Share Hospital payment formulas are evolving to emphasize uncompensated care, and hospitals may experience funding changes as a result.

## Teaser

This cross-sectional study examines characteristics of safety-net hospitals as classified under 3 common definitions based on Medicare Disproportionate Share Hospital payments, Medicaid and uninsured caseload, and uncompensated care costs.

## Introduction

In 2000, the Institute of Medicine defined safety-net hospitals (SNHs) as hospitals that, by mission or mandate, provide care to a substantial share of vulnerable patients regardless of their ability to pay.^1^ According to this definition, any number of public hospitals, academic medical centers, or private hospitals may be designated as SNHs. Hospitals that fall under this broad definition may be similar in that they disproportionately serve vulnerable populations and are under greater financial stress than non-SNHs; however, they also may differ in terms of their organizational structure, size, location, scope of services provided, and degree of financial burden. To date, no consensus exists on how to operationalize the definition of SNHs for research or policy making.

Definitions of SNHs used in empirical studies and government programs attempt to capture key aspects of the safety-net mission. Examples include public ownership, provision of essential services to vulnerable populations, uncompensated care, Medicaid and uninsured inpatient caseload, and the Medicare Disproportionate Share Hospital (DSH) index, which the Centers for Medicare & Medicaid Services (CMS) has historically used to offset uncompensated and unreimbursed care costs for individual hospitals. However, each of these definitions has important limitations and different implications for funding and policy.

First, the safety-net mission is not limited to public ownership. On average, public hospitals tend to care for a larger share of vulnerable populations; however, some SNHs are private, faith-based organizations with a mission to serve these groups.^1^ Larger private hospitals, typically not seen as SNHs, may provide critical SNH services, such as trauma care and specialized intensive care. At the same time, smaller private hospitals that are less likely to provide these services still may be the provider of last resort in their communities.

Second, definitions of uncompensated care,^2,3,4,5,6,7,8^ Medicaid and/or uninsured caseload,^9,10,11,12,13,14,15,16,17,18,19,20,21,22,23,24,25^ and the DSH index^26,27,28,29^ do not cover SNH financial risks and payment shortfalls comprehensively. For example, uncompensated care definitions do not include Medicaid payment shortfalls, whereas definitions based on Medicaid caseloads alone and the historical DSH definition do not account for the burden of caring for the uninsured. Little is known about how common SNH definitions, as applied in research and policy, differ from each other in terms of the types of SNHs they capture.

Understanding the implications of different SNH definitions is particularly important to policy decisions that affect funding for these hospitals. After implementation of the Affordable Care Act (ACA), the formula used to determine how Medicare DSH funds are allocated underwent significant changes.^30^ As ACA-related insurance coverage increased, the pool of uninsured individuals decreased; consequently, the need for DSH funding was expected to decrease. Thus, Medicare DSH payments have been reduced proportionally to decreases in the uninsured population.^31^ However, DSH payment reductions may leave some hospitals financially vulnerable.^31^

As of fiscal year 2018, the Medicare DSH formula—historically only based on Medicaid and Medicare Supplemental Security Income (SSI) inpatient days—now also considers uncompensated care costs.^32^ Critics have argued that the receipt of DSH payments does not always align with the level of uncompensated care provided by hospitals or with provision of essential services to low-income and vulnerable populations.^33,34^ The new formula likely will result in a redistribution of funds among hospitals.^35^ Characteristics of hospitals with a high burden of Medicaid and Medicare SSI inpatient days compared with hospitals with a high burden of uncompensated care costs are largely unknown. A better understanding of these differences would inform policy makers implementing changes to DSH payment policies as well as hospitals that will be affected by these changes.

The goals of the present study were (1) to assess concordance among 3 common SNH definitions based on the historical Medicare DSH index of Medicaid and Medicare SSI inpatient days, Medicaid and uninsured inpatient caseload, and uncompensated care; (2) to evaluate associations between these SNH definitions and hospital organizational characteristics, scope of services provided, and financial attributes; and (3) to shed light on implications of different definitions for policy and funding.

## Methods

### Study Data

This study follows the Strengthening the Reporting of Observational Studies in Epidemiology (STROBE) reporting guidelines for cross-sectional studies.^36^ Data were obtained from 4 primary sources: the Healthcare Cost and Utilization Project State Inpatient Databases^37^ for 47 US states (listed in the Article Information section), CMS Hospital Cost Reports, the American Hospital Association Annual Survey, and Hospital Compare—a public database from CMS that compares the quality of care at Medicare-certified hospitals. We used the State Inpatient Databases, which contain information on all-payer discharges in the United States, to identify study hospitals and ascertain Medicaid and uninsured inpatient caseload. We obtained information on hospital characteristics from the American Hospital Association Annual Survey, information on hospital finances from the CMS Cost Reports, and information on penalties incurred under CMS pay-for-performance programs from Hospital Compare. Because the Healthcare Cost and Utilization Project does not involve human participants, institutional review board approval and written informed consent were not required for this study.

Our analyses focused on fiscal year 2015 and included only hospitals that could be linked across all databases. The 2014-2015 State Inpatient Databases contained 3745 hospitals, of which 2066 non–critical-access hospitals were included in our main analysis. Critical-access hospitals—a designation given by CMS to eligible rural hospitals that typically are financially vulnerable—were excluded from our main analysis comparing SNH definitions because they receive separate funding. Characteristics of these 888 hospitals are provided in the eTable in the Supplement.

### SNH Definitions

We identified alternative SNH definitions through a structured literature review of SNH studies and published guidelines. For our comparisons, we selected definitions in accord with the conceptual framework provided by the Institute of Medicine^1^ and used in research and policy publications. In accord with prior research,^21,24,25,26,27,28,29^ we defined SNHs as those in the top quartile within each state for each of the selected measures described below.

The Medicare DSH index is derived from the CMS Cost Reports and is defined as the number of Medicare SSI inpatient days from total Medicare inpatient days plus the number of Medicaid, non-Medicare inpatient days from total inpatient days.^30^ Before the ACA, the DSH index was the primary measure used to allocate Medicare DSH payments.

Medicaid and the uninsured caseload were derived from Healthcare Cost and Utilization Project data and defined as the percentage of Medicaid and uninsured inpatient stays from all inpatient stays at each hospital. Uninsured stays were identified by state-specific payer codes for indigent care programs, self-pay, and no charge. We chose to include this definition because it is common in the literature^9,10,11,12,13,14,15,16,17,18,19,20,21,22,23,24,25^ and captures concepts measured by the DSH index as described above (ie, Medicaid caseload) as well as concepts measured by uncompensated care (ie, uninsured caseload) as described below.

The cost of uncompensated care, also derived from the CMS Cost Reports, is defined as the cost of charity care plus non-Medicare and nonreimbursable Medicare bad debt.^38^ We calculated the cost of uncompensated care as a percentage of total operating expenses. We chose to include this definition because uncompensated care is becoming increasingly important in determining DSH allocations to hospitals.

### Hospital-Level Variables

Hospital location and structural characteristics obtained from the American Hospital Association included US Census region, urban vs rural location, ownership, size (number of beds), teaching status, health system affiliation, critical access status, and select hospital services.^39^ To better characterize the populations SNHs and non-SNHs serve, we used discharge data from the State Inpatient Databases to calculate each hospital’s percentage of stays by racial/ethnic group, service line, and 4 different median income levels in the patients’ zip code of residence.

Hospital financial variables included the receipt of CMS DSH payments, measures of uncompensated and unreimbursed care, total and operating margins, and CMS penalties. The CMS DSH payments include the value of Medicare DSH payments dispersed directly from CMS to hospitals, as well as whether hospitals received Medicaid DSH payments (yes or no), which are allocated by CMS to states, which then disperse them to hospitals. Because hospitals report Medicaid DSH along with other types of state funding to CMS, the value of Medicaid DSH payments alone cannot be isolated.

Uncompensated care includes charity care costs associated with providing care to the uninsured, as well as non-Medicare and nonreimbursable Medicare bad debt. Bad debt stems from the inability of privately insured patients to pay off high deductibles, as well as debt from services not covered by Medicare either because a patient has Medicare with no secondary insurance or because a patient is dually eligible for Medicare and Medicaid and the state’s Medicaid program does not cover the entire Medicare coinsurance or deductible.^40^ The ACA has resulted in more high-deductible health plans primarily for previously uninsured patients who now have private insurance^41^; therefore, it is important to assess how SNHs and non-SNHs fare in terms of bad debt.

Unreimbursed care, which is measured as the shortfall of revenue relative to cost for Medicaid and other state and local indigent programs, is not slated for consideration in the new DSH payment formula. However, it represents an important component of hospitals’ financial health and may be occurring disproportionately for SNHs vs non-SNHs.

Total and operating profit margins were derived from multiple fields in the CMS Cost Reports and offer important insights into hospitals’ financial health. Penalties incurred by SNHs and non-SNHs under the CMS value-based payment and readmission reduction programs could further affect hospitals’ finances and their ability to provide safety-net services.

### Statistical Analysis

Data were analyzed from March 1 through September 30, 2018, using R, version 3.4.3 (R Project for Statistical Computing). We assessed concordance between SNH definitions using the Cronbach α. We considered values less than 0.50 to indicate no agreement and values equal to or greater than 0.70 to indicate good agreement.^42^ Under each SNH definition, we compared characteristics of SNHs vs non-SNHs. For the financial measures, we present medians instead of means because some distributions of those measures were not normal. We used χ^2^ tests (percentages), unpaired 2-sided *t* tests (means), and Kruskal-Wallis tests (medians) and evaluated levels of statistical significance at *P* < .01 and *P* < .05.

## Results

The 2066 hospitals in this study were distributed across the Northeast (340 [16.5%]), Midwest (587 [28.4%]), South (790 [38.2%]), and West (349 [16.9%]). Each of the 3 SNH definitions generally captured a different set of hospitals (Table 1). Overall, we found no agreement among the 3 alternative SNH definitions (overall Cronbach α = 0.43; 95% CI, 0.38-0.48). Similarly, we found no agreement between definitions based on the DSH index and uncompensated care (Cronbach α = 0.12; 95% CI, 0.03-0.19), and Medicaid and uninsured caseload and uncompensated care (Cronbach α = 0.29; 95% CI, 0.22-0.35). Agreement between DSH index and Medicaid and uninsured caseload-based definitions was low (Cronbach α = 0.56; 95% CI, 0.51-0.60). Only 269 hospitals (13.0%) were identified as SNHs using both the DSH and Medicaid and uninsured caseload definitions; only 187 (9.1%) were identified as SNHs on the Medicaid and uninsured caseload and uncompensated care measures; and only 155 (7.5%) were identified as SNHs on the DSH and uncompensated care measures.

**Table 1.** Concordance Between SNH Definitions^a^

SNH Definition	Hospital Type	No. of Hospitals	No. (%) of Hospitals			
			Medicaid and Uninsured Caseload		Uncompensated Care^b^	
			SNH (n = 487)	Non-SNH (n = 1597)	SNH (n = 523)	Non-SNH (n = 1543)
DSH index	SNH	518	269 (13.0)	249 (12.1)	155 (7.5)	363 (17.6)
	Non-SNH	1548	218 (10.6)	1330 (64.4)	368 (17.8)	1180 (57.1)
Medicaid and uninsured caseload	SNH	487	487 (23.6)	0	187 (9.1)	300 (14.5)
	Non-SNH	1579	0	1579 (76.4)	336 (16.3)	1243 (60.2)

Abbreviations: DSH, Disproportionate Share Hospital; SNH, safety-net hospital.

^a^ The source of the data was our analysis of the Healthcare Cost and Utilization Project State Inpatient Databases and Centers for Medicare & Medicaid Services Cost Reports. Excludes critical-access hospitals, long-term acute care hospitals, and hospitals that are not community nonrehabilitation hospitals. Overall agreement among the 3 definitions: Cronbach α = 0.43 (95% CI, 0.38-0.48). Agreement between the DSH index and Medicaid and uninsured caseload definitions: Cronbach α = 0.56 (95% CI, 0.51-0.60); between the DSH index and uncompensated care definitions: Cronbach α = 0.12 (95% CI, 0.03-0.19); and between Medicaid and uninsured caseload and uncompensated care definitions: Cronbach α = 0.29 (95% CI, 0.22-0.35).

^b^ Defined as bad debt plus charity care.

Table 2 presents characteristics of SNHs and non-SNHs by definition. Across all 3 definitions, SNHs were more likely than non-SNHs to be public hospitals (DSH index, 91 of 518 [17.6%] vs 220 of 1548 [14.2%]; Medicaid and uninsured caseload, 100 of 487 [20.5%] vs 211 of 1579 [13.4%]; uncompensated care, 114 of 523 [21.8%] vs 197 of 1543 [12.8%]). Under the DSH and Medicaid and uninsured caseload definitions, SNHs were more likely than non-SNHs to be larger hospitals with 300 or more beds (264 of 518 [51.0%] vs 311 of 1548 [20.1%] and 158 of 487 [32.4%] vs 417 of 1579 [26.4%], respectively), whereas SNHs identified by uncompensated care were smaller than their non-SNH counterparts (≥300 beds, 95 of 523 [18.2%] vs 480 of 1543 [31.1%]; *P* < .001). The same pattern existed for teaching status (DSH index, 337 of 518 SNHs [65.1%]; Medicaid and uninsured caseload, 229 of 487 SNHs [47.0%]; uncompensated care, 181 of 523 SNHs [34.6%]). Only under the DSH index were SNHs more likely than non-SNHs to be system affiliated (380 of 518 [73.4%] vs 1019 of 1548 [65.8%]). Contrary to the DSH and Medicaid and uninsured caseload definition, SNHs were more likely than non-SNHs to be located in rural areas using the uncompensated care measure (65 of 523 [12.4%] vs 91 of 1543 [5.9%]; *P* < .001). As defined by the DSH index, SNHs provided more essential services than their non-SNH counterparts (eg, alcohol or drug abuse outpatient services, 107 of 518 [20.7%] vs 113 of 1548 [7.3%]; *P* < .001). In contrast, SNHs defined by uncompensated care were less likely than non-SNHs to provide essential services, including services for older adults (220 of 523 [42.1%] vs 756 of 1543 [49.0%]; *P* = .006), neonatal intensive care (126 of 523 [24.1%] vs 563 of 1543 [36.5%]; *P* < .001), and psychiatric outpatient services (156 of 523 [29.8%] vs 555 of 1543 [36.0%]; *P* = .01).

**Table 2.** Characteristics of Study Hospitals by Type of SNH Definition^a^

Characteristic	SNH Definition					
	DSH Index		Medicaid and Uninsured Caseload		Uncompensated Care	
	SNH (n = 518)	Non-SNH (n = 1548)	SNH (n = 487)	Non-SNH (n = 1579)	SNH (n = 523)	Non-SNH (n = 1543)
**Hospital Characteristics**						
Ownership, No. (%)						
Public	91 (17.6)	220 (14.2)	100 (20.5)^b^	211 (13.4)^b^	114 (21.8)^b^	197 (12.8)^b^
Private, nonprofit	380 (73.4)	1164 (75.2)	343 (70.4)^c^	1201 (76.1)^c^	385 (73.6)	1159 (75.1)
Private, for profit	47 (9.1)	164 (10.6)	44 (9.0)	167 (10.6)	24 (4.6)^b^	187 (12.1)^b^
Hospital size, No. of beds (%)						
6-99	38 (7.3)^b^	598 (38.6)^b^	146 (30.0)	490 (31.0)	200 (38.2)^b^	436 (28.3)^b^
100-299	216 (41.7)	639 (41.3)	183 (37.6)	672 (42.6)	228 (43.6)	627 (40.6)
≥300	264 (51.0)^b^	311 (20.1)^b^	158 (32.4)^b^	417 (26.4)^b^	95 (18.2)^b^	480 (31.1)^b^
Teaching hospital, No. (%)	337 (65.1)^b^	543 (35.1)^b^	229 (47.0)^c^	651 (41.2)^c^	181 (34.6)^b^	699 (45.3)^b^
System affiliated, No. (%)	380 (73.4)^a^	1019 (65.8)^a^	335 (68.8)	1064 (67.4)	343 (65.6)	1056 (68.4)
Location, No. (%)						
Large metropolitan	248 (47.9)^b^	637 (41.1)^b^	191 (39.2)	694 (44.0)	209 (40.0)	676 (43.8)
Small metropolitan	195 (37.6)^b^	482 (31.1)^b^	141 (29.0)^c^	536 (33.9)^c^	149 (28.5)^c^	528 (34.2)^c^
Micropolitan	60 (11.6)^b^	288 (18.6)^b^	114 (23.4)^b^	234 (14.8)^b^	100 (19.1)	248 (16.1)
Rural (noncore)	15 (2.9)^b^	141 (9.1)^b^	41 (8.4)	115 (7.3)	65 (12.4)^b^	91 (5.9)^b^
Region, No. (%)						
Northeast	85 (16.4)	255 (16.5)	59 (12.1)^b^	281 (17.8)^b^	85 (16.3)	255 (16.5)
Midwest	148 (28.6)	439 (28.4)	148 (30.4)	439 (27.8)	148 (28.3)	439 (28.5)
South	194 (37.5)	596 (38.5)	190 (39.0)	600 (38.0)	199 (38)	591 (38.3)
West	91 (17.6)	258 (16.7)	90 (18.5)	259 (16.4)	91 (17.4)	258 (16.7)
Select hospital services, No. (%)						
Alcohol/drug abuse outpatient	107 (20.7)^b^	113 (7.3)^b^	71 (14.6)^b^	149 (9.4)^b^	60 (11.5)	160 (10.4)
Alzheimer disease center	76 (14.7)^b^	81 (5.2)^b^	45 (9.2)	112 (7.1)	33 (6.3)	124 (8.0)
Services for older adults	318 (61.4)^b^	658 (42.5)^b^	233 (47.8)	743 (47.1)	220 (42.1)^b^	756 (49.0)^b^
HIV/AIDS	286 (55.2)^b^	480 (31.0)^b^	203 (41.7)^c^	563 (35.7)^c^	183 (35.0)	583 (37.8)
Indigent care clinic	198 (38.2)^b^	321 (20.7)^b^	153 (31.4)^b^	366 (23.2)^b^	121 (23.1)	398 (25.8)
Neonatal intensive care unit	312 (60.2)^b^	377 (24.4)^b^	204 (41.9)^b^	485 (30.7)^b^	126 (24.1)^b^	563 (36.5)^b^
Psychiatric acute care unit	337 (65.1)^b^	546 (35.3)^b^	243 (49.9)^b^	640 (40.5)^b^	218 (41.7)	665 (43.1)
Psychiatric outpatient	276 (53.3)^b^	435 (28.1)^b^	212 (43.5)^b^	499 (31.6)^b^	156 (29.8)^c^	555 (36.0)^c^
Psychiatric emergency	353 (68.1)^b^	636 (41.1)^b^	256 (52.6)^c^	733 (46.4)^c^	246 (47.0)	743 (48.2)
Trauma center	329 (63.5)^b^	616 (39.8)^b^	266 (54.6)^b^	679 (43.0)^b^	248 (47.4)	697 (45.2)
**Patient Characteristics**						
Racial/ethnic minority, mean (SD), %	39.6 (25.3)^b^	23.4 (21.3)^b^	36.8 (26.0)^b^	24.3 (21.6)^b^	31.1 (26.1)^b^	26.2 (22.3)^b^
Community income, mean (SD), %						
Quartile 1 (lowest)	37.8 (23.1)^b^	24.5 (23.8)^b^	38.5 (26.5)^b^	24.2 (22.4)^b^	31.3 (28.0)^b^	26.7 (22.8)^b^
Quartile 2	27.3 (16.0)^b^	30.4 (21.6)^b^	30.4 (20.5)	29.3 (20.4)	30.9 (22.8)	29.1 (19.5)
Quartile 3	21.0 (13.2)^b^	25.2 (17.9)^b^	19.9 (17.0)^b^	25.6 (16.7)^b^	23.6 (19.6)	24.3 (16.0)
Quartile 4 (highest)	13.9 (13.3)^b^	19.9 (22.1)^b^	11.1 (14.6)^b^	20.9 (21.5)^b^	14.1 (17.3)^b^	19.8 (21.2)^b^
Service line, mean (SD), %						
Maternal/neonatal	24.0 (12.4)^b^	20.3 (15.3)^b^	27.0 (14.5)^b^	19.2 (14.2)^b^	21.5 (15.1)	21.1 (14.6)
Mental health	8.2 (10.0)^b^	4.8 (7.8)^b^	8.3 (12.2)^b^	4.7 (6.7)^b^	6.9 (10.6)^b^	5.2 (7.7)^b^
Injury	4.8 (3.0)^b^	4.0 (2.5)^b^	4.3 (3.0)	4.1 (2.5)	4.1 (2.8)	4.2 (2.6)
Surgical	17.4 (6.6)^b^	20.9 (18.6)^b^	14.3 (8.8)^b^	22.0 (18.0)^b^	13.8 (8.9)^b^	22.1 (17.9)^b^
General medical	45.6 (10.4)^b^	50.1 (18.1)^b^	46.1 (12.3)^b^	50.0 (17.7)^b^	53.6 (16.8)^b^	47.4 (16.3)^b^

Abbreviations: DSH, Disproportionate Share Hospital; SNH, safety-net hospital.

^a^ Source was our analysis of the Healthcare Cost and Utilization Project State Inpatient Databases, Centers for Medicare & Medicaid Services Cost Reports, and the American Hospital Association Annual Survey. Excludes critical-access hospitals, long-term acute care hospitals, and hospitals that are not community nonrehabilitation hospitals. Uncompensated care is defined as bad debt plus charity care. Percentages have been rounded and may not total 100.

^b^ *P* < .01, χ^2^ test or unpaired 2-tailed *t* test of the difference between percentages or means, respectively, across SNHs and non-SNHs.

^c^ *P* < .05, χ^2^ test or *t* test of the difference between percentages or means, respectively, across SNHs and non-SNHs.

Under each definition, SNHs were more likely than non-SNHs to treat a greater percentage of racial/ethnic minorities (mean [SD] for DSH index, 39.6% [25.3%] vs 23.4% [21.3%]; Medicaid and uninsured caseload, 36.8% [26.0%] vs 24.3% [21.6%]; uncompensated care, 31.1% [26.1%] vs 26.2% [22.3%]), patients from communities in the lowest income quartile (mean [SD] for DSH index 37.8% [23.1%] vs 24.5% [23.8%]; Medicaid and uninsured caseload, 38.5% [26.5%] vs 24.2% [22.4%]; uncompensated care, 31.3% [28.0%] vs 26.7% [22.8%]), and patients receiving mental health care (mean [SD] for DSH index, 8.2% [10.0%] vs 4.8% [7.8%]; Medicaid and uninsured caseload, 8.3% [12.2%] vs 4.7% [6.7%]; uncompensated care, 6.9% [10.6%] vs 5.2% [7.7%]). Under the DSH and Medicaid and uninsured caseload definitions, SNHs also were more likely to have a greater mean (SD) percentage of maternal/neonatal stays than non-SNHs (24.0% [12.4%] vs 20.3% [15.3%] and 27.0% [14.5%] vs 19.2% [14.2%], respectively; *P* < .001). In addition, under the DSH definition, SNHs treated more patients with injuries than non-SNHs (mean [SD], 4.8% [3.0%] vs 4.0% [2.5%]; *P* < .001). In contrast, SNHs and non-SNHs as defined by uncompensated care had a similar mean (SD) percentage of stays for maternal/neonatal (21.5% [15.1%] vs 21.1% [14.6%]; *P* = .54) and injury-related (4.1% [2.8%] vs 4.2% [2.6%]; *P* = .75) services.

Table 3 presents hospital financial characteristics under each definition. Median (interquartile range [IQR]) Medicare DSH payments were higher for SNHs than non-SNHs under the DSH ($854 263 [$470 661-$1 219 269] vs $176 511 [$17 484-$510 288]; *P* < .001) and Medicaid and uninsured caseload ($241 196 [$112 665-$746 398] vs $236 024 [$20 312-$709 376]; *P* = .002) definitions but were similar for SNHs and non-SNHs under the uncompensated care definition ($221 274 [$69 674-$691 961] vs $238 882 [$25 864-$728 230]; *P* = .35). Medicare DSH payments per $1000 of operating expenses remained higher for SNHs than non-SNHs under each definition. However, although median (IQR) DSH payments were more than 2 times higher for SNHs than non-SNHs as defined by the DSH index ($5.4 [$3.9-$7.5] vs $2.1 [$0.7-$3.4] per $1000 of operating expenses; *P* < .001), they were only 15% higher for SNHs than non-SNHs as defined by uncompensated care ($3.0 [$1.6-$5.0] vs $2.6 [$1.0-$4.4] per $1000 of operating expenses; *P* < .001). The percentage of hospitals that received Medicaid DSH payments also was higher for SNHs than non-SNHs under the DSH (439 of 518 [84.7%] vs 1016 of 1548 [65.6%]) and Medicaid and uninsured caseload (394 of 487 [80.9%] vs 1061 of 1579 [67.2%]) definitions (*P* < .001), but the percentages for SNHs and non-SNHs were similar under the uncompensated care definition (383 of 523 [73.2%] vs 1072 of 1543 [69.5%]; *P* = .10).

**Table 3.** Financial Characteristics of SNHs and Non-SNHs by Type of SNH Definition^a^

Characteristic	SNH Definition					
	DSH Index		Medicaid and Uninsured Caseload		Uncompensated Care	
	SNH (n = 518)	Non-SNH (n = 1548)	SNH (n = 487)	Non-SNH (n = 1579)	SNH (n = 523)	Non-SNH (n = 1543)
CMS payments, median (IQR), $						
Medicare DSH payment	854 263 (470 661 to 1 219 269)^b^	176 511 (17 484 to 510 288)^b^	241 196 (112 665 to 746 398)^b^	236 024 (20 312 to 709 376)^b^	221 274 (69 674 to 691 961)	238 882 (25 864 to 728 230)
Medicare DSH payment per $1000 of total operating expenses	5.4 (3.9 to 7.5)^b^	2.1 (0.7 to 3.4)^b^	4.1 (2.4 to 6.4)^b^	2.4 (0.9 to 4.1)^b^	3.0 (1.6 to 5.0)^b^	2.6 (1.0 to 4.4)^b^
Received Medicaid DSH payment, No. (%)	439 (84.7)^b^	1016 (65.6)^b^	394 (80.9)^b^	1061 (67.2)^b^	383 (73.2)	1072 (69.5)
Components of uncompensated and unreimbursed costs, median (IQR), $						
Bad debt expense per $1000 of total operating expenses^c^	15.3 (8.4 to 28.2)	15.2 (7.7 to 26.8)	19.1 (10.1 to 31.9)^b^	14.1 (7.5 to 25.6)^b^	27.1 (15.5 to 44.3)^b^	12.8 (6.7 to 21.6)^b^
Charity care cost per $ 1000 of total operating expenses	12.6 (5.8 to 25.0)^b^	10.5 (4.4 to 21.1)^b^	13.2 (5.6 to 25.0)^b^	10.4 (4.5 to 21.3)^b^	19.9 (9.3 to 34.1)^b^	9.1 (4.0 to 18.7)^b^
Unreimbursed cost per $1000 of total operating expenses^d^	23.9 (7.4 to 46.2)	25.4 (10.9 to 45.8)	27.5 (6.5 to 50.2)	24.6 (10.4 to 44.7)	32.6 (12.4 to 55.4)^b^	23.6 (9.0 to 42.7)^b^
Profit margins, median (IQR), %						
Net profit margin^e^	5.1 (1.2 to 9.9)	5.7 (0.8 to 11.4)	5.4 (0.4 to 10.6)	5.6 (1.0 to 11.0)	4.7 (0 to 9.9)^b^	5.8 (1.2 to 11.2)^b^
Operating profit margin^f^	1.2 (−4.6 to 7.1)	2.1 (−4.4 to 9.1)	0.9 (−7.1 to 7.1) ^b^	2.2 (−3.9 to 8.9) ^b^	0.3 (−8.0 to 7.2)^b^	2.3 (−3.9 to 8.9)^b^
Value-based purchasing bonuses and penalties, No. (%)						
Bonus	214 (41.3)	711 (45.9)	191 (39.2)^b^	734 (46.5)^b^	216 (41.3)	709 (45.9)
No adjustment	23 (4.4)^b^	217 (14.0)^b^	51 (10.5)	189 (12.0)	68 (13.0)	172 (11.1)
Penalty	281 (54.2)^b^	620 (40.1)^b^	245 (50.3)^b^	656 (41.5)^b^	239 (45.7)	662 (42.9)
Hospital readmission reduction program penalties, No. (%)						
High penalty (≥1%)	32 (6.2)	82 (5.3)	34 (7.0)	80 (5.1)	33 (6.3)	81 (5.2)
Low penalty (<1%)	365 (70.5)^b^	874 (56.5)^b^	302 (62.0)	937 (59.3)	318 (60.8)	921 (59.7)
No penalty	121 (23.4)^b^	592 (38.2)^b^	151 (31.0)	562 (35.6)	172 (32.9)	541 (35.1)

Abbreviations: CMS, Centers for Medicare & Medicaid Services; DSH, Disproportionate Share Hospital; IQR, interquartile range; SNH, safety-net hospital.

^a^ Source was the authors’ analysis of the Healthcare Cost and Utilization Project State Inpatient Databases, CMS Cost Reports, and Hospital Compare. Excludes critical-access hospitals, long-term acute care hospitals, and hospitals that are not community nonrehabilitation hospitals.

^b^ *P* < .01, χ^2^ test or Kruskal-Wallis test of the difference between percentages or distributions, respectively, across SNHs and non-SNHs.

^c^ Includes non-Medicare and nonreimbursable Medicare bad debt.

^d^ Includes operating costs associated with Medicaid, Children’s Health Insurance Program, and county and state indigent care programs.

^e^ Calculated as net income or loss divided by the sum of net patient revenue plus total other income.

^f^ Calculated as net patient revenue minus the sum of total operating expenses plus other operating income (excluding government appropriations and unitemized miscellaneous income) divided by the sum of net patient revenue plus other operating income (excluding government appropriations and unitemized miscellaneous income).

Among the 3 definitions, only the one based on uncompensated care burden revealed remarkable differences between SNHs and non-SNHs for all 3 components of uncompensated and unreimbursed costs per operating expenses. Under this definition, median (IQR) bad debt ($27.1 [$15.5-$44.3] vs $12.8 [$6.7-$21.6] per $1000 of operating expenses; *P* < .001) and charity care ($19.9 [$9.3-$34.1] vs $9.1 [$4.0-$18.7] per $1000 of operating expenses; *P* < .001) costs for SNHs were about twice as high as those for non-SNHs; median (IQR) unreimbursed costs for SNHs were 38% higher than for non-SNHs ($32.6 [$12.4-$55.4] vs $23.6 [$9.0-$42.7] per $1000 of operating expenses; *P* < .001). Differences between SNHs and non-SNHs for median (IQR) bad debt under the DSH ($15.3 [$8.4-$28.2] vs $15.2 [$7.7-$26.8] per $1000 of operating expenses; *P* = .32) and Medicaid and uninsured caseload ($19.1 [$10.1-$31.9] vs $14.1 [$7.5-$25.6] per $1000 of operating expenses; *P* < .001) definitions were not as large in magnitude, nor were differences in median (IQR) charity care costs ($12.6 [$5.8-$25.0] vs $10.5 [$4.4-$21.1] per $1000 of operating expenses [*P* < .001] and $13.2 [$5.6-$25.0] vs $10.4 [$4.5-$21.3] per $1000 of operating expenses [*P* = .001]) or unreimbursed costs ($23.9 [$7.4-$46.2] vs $25.4 [$10.9-$45.8] per $1000 of operating expenses [*P* = .21] and $27.5 [$6.5-$50.2] vs $24.6 [$10.4-$44.7] per $1000 of operating expenses [*P* = .45], respectively).

Safety-net hospitals defined by uncompensated care burden had lower median (IQR) net total (4.7% [0%-9.9%] vs 5.8% [1.2%-11.2%]; *P* = .003) and operating (0.3% [−8.0% to 7.2%] vs 2.3% [−3.9% to 8.9%]; *P* < .001) profit margins than their non-SNH counterparts, whereas differences between SNH and non-SNH profit margins generally were not statistically significant under the other 2 definitions. Safety-net hospitals under the uncompensated care definition had the lowest median (IQR) profit margins of any group of hospitals we examined (0.3% [−8.0% to 7.2%]), with 25% of hospitals falling below −8.0%. Finally, under the DSH and Medicaid and uninsured caseload measures, SNHs were more likely than non-SNHs to experience penalties under CMS pay-for-performance programs (eg, penalty under the value-based purchasing program, 281 of 518 [54.2%] vs 620 of 1548 [40.1%] for DSH and 245 of 487 [50.3%] vs 656 of 1579 [41.5%] for Medicaid and uninsured caseload; *P* < .01).

## Discussion

In this study, by leveraging all-payer discharge data from the Healthcare Cost and Utilization Project as well as hospital-level information from the American Hospital Association and CMS, we found limited concordance between current definitions of SNHs. Despite some overlap, each definition appears to capture a set of hospitals with different organizational and financial characteristics, rural or urban location, and breadth of services provided. Although this finding is consistent with previous research,^43^ our study also underscores the consequences of using different types of definitions for SNH funding. This difference is particularly salient given current changes in funding policy.

Compared with SNHs identified by other definitions, SNHs identified by a high DSH index are larger, urban, teaching hospitals and more often part of a health system. They tend to provide a broad array of services important to the safety-net mission. Financially speaking, they have lower uncompensated and unreimbursed costs and relatively better operating margins than SNHs identified by other definitions. Thus, although these hospitals appear more vulnerable than their non-SNH counterparts, they may be less financially vulnerable than SNHs under other definitions.

At the other end of the spectrum, SNHs identified by uncompensated care costs are smaller, nonteaching institutions less often affiliated with health systems, more often located in rural areas, and providing fewer specialized services related to vulnerable populations. These hospitals have lower Medicare DSH payments and the lowest profit margins, likely because uncompensated and unreimbursed costs constitute a greater proportion of operating expenses than for other types of SNHs. Thus, these hospitals appear more vulnerable financially. In turn, higher financial vulnerability may interfere with their ability to expand services and provide comprehensive care to vulnerable populations in the communities they serve, for which they may be the provider of last resort.

Finally, in line with previous research,^27^ we found that SNHs, particularly those identified by the DSH index and Medicaid and uninsured caseload, were more likely than non-SNHs to incur CMS penalties. These findings fit into the ongoing debate regarding the disproportionate risk to SNHs posed by CMS value-based policies, leading to the introduction of a new socioeconomic status adjustment to CMS’s method of calculating penalties for excess readmissions.^44^

Our findings are particularly relevant considering CMS’s changes to the Medicare DSH payment formula. Before the ACA, supplemental payments aimed at subsidizing safety-net care were allocated based on the hospital’s share of patients covered by Medicare SSI and Medicaid, ignoring the burden of uncompensated and unreimbursed care. In response to criticism that DSH payments were excessive and not necessarily reaching hospitals providing the most uncompensated or unreimbursed care,^45^ the ACA introduced significant changes to the DSH payment formula. First, total available DSH payments are reduced in accord with a declining uninsured population. Second, CMS adjusted its DSH individual payment formula to place more weight on the amount of uncompensated care a hospital provides. The current formula is the result of 3 factors: DSH payments that would otherwise be made under the old DSH method; the percentage of change in the national estimate of uninsured individuals younger than 65 years; and the hospital’s share of uncompensated care costs relative to aggregate uncompensated care costs across all DSH hospitals.^30^

Although the new formula now accounts for uncompensated care, it is important to understand nuances in its implementation. For instance, the new formula may still favor larger DSH hospitals, because these hospitals likely have larger shares of total uncompensated care costs across hospitals. In addition, the formula defines uncompensated care as charity care plus bad debt but does not account for other unreimbursed costs, such as Medicaid payment shortfalls. Finally, our study suggests that bad debt constitutes a larger proportion of uncompensated care costs than charity care. Future research may explore components of bad debt (ie, non-Medicare and nonreimbursable Medicare debt) to understand how each may be driving payments.

In contrast, we evaluated an SNH definition based on hospitals’ uncompensated care costs per $1000 of operating expenses, which in accord with past research^4,5,6,7,8^ account for the burden of uncompensated care on each hospital’s finances. Thus, like the DSH index, which uses total inpatient days as a denominator, our measure divides by operating expenses. We found that hospitals identified by uncompensated care burden are the most financially vulnerable SNH subgroup. Efforts to refine the DSH payment formula to target financially vulnerable hospitals may miss smaller hospitals with a high share of total uncompensated care costs, which also appear to have high shares of unreimbursed care costs among patients with Medicaid.

Although the present study only evaluates established SNH definitions and does not actually simulate the newly released Medicare DSH payment formula, it provides an important baseline for research and policy. Future research should compare existing and new DSH payments and evaluate the effects of the new formula and expected funding shifts on hospitals’ financial well-being and ability to perform key safety-net functions.

Although SNHs appear to serve higher proportions of minorities and individuals with low incomes than non-SNHs across all definitions, this differential was greatest under the DSH and Medicaid and uninsured caseload measures. Given the lack of consensus, researchers and policy makers conducting disparities studies should use the particular definitions that best fit their aims, conceptually and empirically, and keep heterogeneity across definitions in mind.

### Limitations

This study has several limitations. Two of our SNH definitions (the DSH index and uncompensated care) rely on the validity of data in the CMS Cost Reports. Public concerns regarding variation in how hospitals report charity care and bad debt on the S-10 worksheet caused a delay in the use of uncompensated care to allocate DSH payments.^46^ Although the quality of data may be improving,^46^ our analysis is based on what hospitals reported in fiscal year 2015, and our results likely reflect evolving hospital reporting practices. We chose fiscal year 2015 because at the time the study was conducted, this was one of the latest years available that reflected implementation of the ACA (January 2014) and retained the *International Classification of Diseases, Ninth Revision*, coding system (the *International Statistical Classification of Diseases and Related Health Problems, Tenth Revision*, was implemented in October 2015).

## Conclusions

Different definitions appear to capture a heterogeneous set of SNHs in terms of their organizational and financial characteristics, location, and services provided. This variety suggests that each SNH definition highlights different dimensions of the safety-net mission. Payment formulas based on multiple measures may more successfully distribute funds to different types of SNHs. Disproportionate Share Hospital payment formulas appear to be moving in this direction by emphasizing uncompensated care costs, but other potentially important measures, such as unreimbursed care costs and uninsured caseloads, are not included. We believe future research should evaluate how Medicare and Medicaid DSH policies ultimately redistribute funds across different types of hospitals. Future payment policies may need to account for differences between SNH definitions so that resources can be distributed more equitably across a diverse set of SNHs to support essential aspects of the safety-net mission.

## Notes

Supplement. **eTable.** Characteristics of Critical-Access Hospitals (CAHs)
