# Supplementary material for: Comparison of 3 Safety-Net Hospital Definitions and Association With Hospital Characteristics
Source: JAMA Netw Open. 2019 Aug 7;2(8):e198577. doi: 10.1001/jamanetworkopen.2019.8577 (PMC6686776; doi:10.1001/jamanetworkopen.2019.8577)
Supplement: Supplement. — eTable. Characteristics of Critical-Access Hospitals (CAHs) [file jamanetwopen-2-e198577-s001.pdf]

Supplementary Online Content

Popescu I, Fingar KR, Cutler E, Guo J, Jiang HJ. Comparison of 3 safety-net hospital definitions and association with hospital characteristics. *JAMA Netw Open.* 2019;2(8):e198577. doi:10.1001/jamanetworkopen.2019.8577

eTable. Characteristics of Critical-Access Hospitals (CAHs)

This supplementary material has been provided by the authors to give readers additional information about their work.

© 2019 Popescu I et al. *JAMA Network Open*.

eTable. Characteristics of Critical-Access Hospitals (CAHs)[a,b]

Characteristic	Hospitals (N = 888), %
**Institutional characteristics, % of CAHs**	
Ownership	
Public	39.8
Private, nonprofit	56.2
Private, for profit	4.1
Bed size	
6-99 beds	94.6
100-299 beds	5.4
300+ beds	0.0
Teaching hospital	2.7
System affiliated	48.6
Location	
Large metropolitan	4.7
Small metropolitan	13.4
Micropolitan	17.3
Rural (noncore)	64.5
Region	
Northeast	4.3
Midwest	53.4
South	25.5
West	16.9
**Select hospital services, %**	
Alcohol/drug abuse outpatient	2.4
Alzheimer's center	3.4
Services for older adults	31.6
HIV/AIDS	2.9
Indigent care clinic	10.1
Neonatal intensive care unit	1.5
Psychiatric acute care unit	6.3
Psychiatric outpatient	14.6
Psychiatric emergency services	12.0
Trauma center	41.9
**Patient characteristics, mean % of discharges at CAHs**	
Racial/ethnic minority	12.1
Community income	
Quartile 1 (lowest)	34.0
Quartile 2	39.6
Quartile 3	21.6
Quartile 4 (highest)	4.8

© 2019 Popescu I et al. *JAMA Network Open*.

Characteristic	Hospitals (N = 888), %
Service line	
Maternal/neonatal	12.3
Mental health	2.4
Injury	3.4
Surgical	5.4
Medical	76.5
**Financial characteristics**	
CMS payments	
Medicare DSH payment, median $	N/A
Medicare DSH payment per $1,000 of total operating expenses, median $	N/A
Received Medicaid DSH, %	51.2
Components of uncompensated and underreimbursed care	
Charity care per $1,000 of total operating expenses, median $	7.2
Bad debt expense per $1,000 of total operating expenses, median $	25.1
Unreimbursed cost per $1,000 of total operating expenses, median $	22.7
Profit margins	
Net profit margin, median %	3.2
Operating profit margin, median %	-4.0
Value-based purchasing bonuses/penalty, %	
Bonus	0.0
No adjustment	100.0
Penalty	0.0
Hospital readmission reduction program penalty, %	
High penalty (≥1%, ≤3%)	0.0
Low penalty (<1%)	0.0
No penalty	100.0

Abbreviations: AIDS, Acquired Immunodeficiency Syndrome; Centers for Medicare & Medicaid; DSH, Disproportionate Share Hospital; HIV, Human Immunodeficiency Virus; SNH, safety-net hospital.

[a] Source was the authors' analysis of the Healthcare Cost and Utilization Project State Inpatient Databases, Centers for Medicare & Medicaid Services Cost Reports, the American Hospital Association (AHA) Annual Survey, and Hospital Compare.

[b] Long-term acute care hospitals and hospitals that are not community nonrehabilitation hospitals. Bad debt includes non-Medicare and nonreimbursable Medicare bad debt. Unreimbused costs include those from Medicaid, Children's Health Insurance Program, and county and state indigent care programs. Net profit margin calculated as net income or loss / (net patient revenue + total other income). Operating profit margin calculated as net patient revenue – total operating expenses + other operating income excluding government appropriations and unitemized miscellaneous income) / (net patient revenue + other operating income excluding government appropriations and unitemized miscellaneous income).

© 2019 Popescu I et al. *JAMA Network Open*.
